# Risk of Second Primary Malignancies Based on the Histological Subtypes of Colorectal Cancer

**DOI:** 10.3389/fonc.2021.650937

**Published:** 2021-03-10

**Authors:** Meijuan Wu, Mengxi Huang, Chenglong He, Cheng Chen, Huiyu Li, Jing Wang, Mengyan Liu, Gongbo Fu, Zengjie Lei, Xiaoyuan Chu

**Affiliations:** ^1^Department of Medical Oncology, Affiliated Jinling Hospital, Medical School of Nanjing University, Nanjing, China; ^2^Department of Medical Oncology, Jinling Hospital, The First School of Clinical Medicine, Southern Medical University, Nanjing, China

**Keywords:** colorectal cancer, second primary malignancies, histological subtypes, classical adenocarcinoma, mucinous adenocarcinoma, signet-ring cell carcinoma, SEER database

## Abstract

**Background:** Previous studies have revealed an increased risk of second primary malignancies (SPMs) after colorectal cancer (CRC); however, no previous investigation has quantified differences in the risk of SPMs based on the histological subtypes of first primary CRC.

**Methods:** Patients diagnosed with first primary CRC between 2000 and 2011 were identified from the Surveillance, Epidemiology, and End Results cancer registries. The patients were divided into three cohorts: classical adenocarcinoma (CA), mucinous adenocarcinoma (MA), and signet-ring cell carcinoma (SRCC). Standardized incidence ratios were calculated to assess the risk of SPMs among the patients.

**Results:** Overall risk of SPMs was significantly higher among patients with three histological subtypes of CRC than in the general population. The risk of esophagus cancer was significantly increased in SRCC. The risk of small intestine, colon and rectum, and corpus uteri cancers was high in three histological subtypes, with the highest risk observed in SRCC, followed by MA. Increased risks of second stomach, uterus, urinary bladder, kidney, and thyroid cancers were only observed in CA patients, while increased risk of second renal pelvis cancer was limited to MA patients. Furthermore, the high overall risk of SPMs in CA patients persisted regardless of clinicopathological factors. After surgery combined with chemotherapy treatment, CA patients were more prone to developing second small intestine, colon and rectum cancers than those treated with surgery only. A lower second prostate cancer risk was observed in rectal CA patients treated with surgery combined with radiotherapy than in patients treated with surgery only.

**Conclusion:** The present study revealed that the risk of developing SPMs after CRC varied based on the histological subtypes of the first primary CRC. Although the mechanisms underlying the observed patterns of SPM risk remain unknown, the study provided insights into future cancer surveillance based on the histological subtypes of CRC.

## Introduction

Colorectal cancer (CRC) is the third most widespread cause of cancer-related deaths in both men and women in the United States, and ranks second when men and women are combined (1). According to Survivorship Statistics released by the American Cancer Society, it was estimated that more than 1.5 million survivors in the United States were living with a previous CRC diagnosis in 2019 (2). Advances in early detection and treatment of CRC have contributed to enhancements in CRC prognosis, which could be the reason for the increase in the population of CRC survivors (2). Therefore, a long life exposes these survivors to long-term health concerns, including the development of second primary malignancies (SPMs). Several population-based studies have demonstrated an increased risk of developing SPMs after a previous diagnosis of CRC compared with the general population (3–7). Although the underlying mechanisms remain unknown, the increased risk could be associated with shared genetic or environmental risk factors for different malignancies or a side effect of previous treatment for CRC.

Most CRCs are adenocarcinomas, including three key comprehensively studied subtypes: classical adenocarcinoma (CA), mucinous adenocarcinoma (MA), and signet-ring cell carcinoma (SRCC) (8). MA and SRCC are rare subtypes of CRC and exist distinct characteristics from CA, including a younger age of onset, more advanced stage, and increased possibility of lymphatic invasion and perineural infiltration (8–12). Therefore, it is plausible that the risk and distribution of SPMs in different histological subtypes of CRC could differ.

In addition, the treatment of CRC varies with the stage at diagnosis; however, the most widespread treatment administered is surgery, with additional therapy including systemic chemotherapy and radiation therapy (radiation therapy is used more often in rectal cancer than in colon cancer cases) administered either in the neoadjuvant or adjuvant setting (13). The treatment patterns could influence the risk of SPMs as chemotherapy and radiotherapy are carcinogenic and have been demonstrated to increase the risk of SPMs at various sites, including lung, stomach, colorectal sites and the bladder (14–16).

To the best of our knowledge, this is the first systematic retrospective study focusing on risk and distribution of SPMs after CRC based on the histological subtypes, with a large sample of CRC patients obtained from the Surveillance, Epidemiology, and End Results (SEER) database. Understanding the patterns could provide further insights into the epidemiology of CRC and guide clinical decisions regarding surveillance and adjuvant treatment after CRC.

## Patients and Methods

### Data Source

Data used in the present study were extracted from the National Cancer Institute's Surveillance, Epidemiology, and End Results (SEER) program database, which contained information from population-based cancer registries on patient demographics, cancer incidence, treatment, and outcomes (https://seer.cancer.gov). The database we selected was SEER 18 regs, excluding AK Custom Data (with additional treatment fields), which was submitted in November 2018 (2000–2016). To distinguish second primary malignancies from recurrences and metastases, SEER registrars follow a series of coding rules considering site, histology, timing and whether anastomotic lesions have mucosal involvement.

### Patient Selection

Patients aged 20–79 years, who had been initially diagnosed with CRC presenting malignant behavior between January 2000 and December 2011 were included in the study. Patients were followed-up for at least 5 years to determine the risk of developing SPMs. Patients with reports presented in the form of death certificates or autopsy only were not enrolled, as were those without pathologically confirmed diagnoses. We further selected patients diagnosed with three histological subtypes of CRC: CA (Codes: 8140–8147, 8210–8213, 8220–8221, 8255, 8260–8263, 8310-8323), MA (Codes: 8480–8481), and SRCC (Codes: 8490) based on ICD-O-3 codes. Patients diagnosed as other histological subtypes were excluded. Detailed corresponding descriptions of the three histological subtypes of CRC are presented in Supplementary Table 1. To enhance the validity and authenticity of the present study, patients with missing values on crucial covariates such as race, grade and SEER summary stage were excluded. Additional exclusion criteria included patients with performance of surgery noted death certificate/autopsy or unknown operation. To identify the location of tumors, tumor sites such as appendix, overlapping lesions of colon or colon not otherwise specified (NOS) were excluded. Finally, patients who were followed <6 months or were diagnosed with SPMs within the first 6 months after initial CRC were not enrolled to exclude synchronous primary malignancies. Overall, the enrolled CRC patients were divided into three cohorts: classical adenocarcinoma (CA), mucinous adenocarcinoma (MA), and signet-ring cell carcinoma (SRCC) cohorts. A flowchart of the selection criteria of patients is presented in Supplementary Figure 1.

### Statistical Analysis

Standardized incidence ratios (SIRs) were calculated using multiple primary-standardized incidence ratio (MP-SIR) sessions of SEER^*^Stat version 8.3.8 (SEER Program, National Cancer Institute). The SIR, also known as relative risk, represents the ratio of the observed number of second cancers to the expected number of cancers based on the US general population, with adjustment for race, sex, age and calendar year by the specific stratified person-time variable accrued from the CRC cohort. Data on cancer incidence among the general population were retrieved from the SEER 18 regs, excluding AK Custom Data (with additional treatment fields), which was submitted in November 2018. More detailed information regarding the SEER^*^Stat software and the methods to calculate the SIRs is available on the SEER-registry website (https://seer.cancer.gov/resources/). We compared SIRs between CA, MA, and SRCC cases for each multiple primary cancer site using poisson regression. SIRs for subgroup analyses were further stratified by sex, race, age at diagnosis, year at diagnosis, grade, tumor site, and SEER summary stage of the first primary CRC. We compared SIRs between different treatments received by patients (surgery only vs. surgery combined with chemotherapy, and surgery only vs. surgery combined with radiotherapy) in CA, MA, and SRCC cases using poisson regression. Demographic and clinical features were analyzed using a Chi-square test. R statistical software version 3.5.0 (Bell Laboratories, Murray Hill, New Jersey, United States) was used to perform Chi-square test and poisson regression. Evaluation of the confidence intervals (CIs) of SIRs was used to determine any overlap. A two-sided *p* < 0.05 was considered statistically significant for all analyses.

## Results

### Patient Characteristics

Patient demographics grouped based on the histological subtypes of CRC are summarized in Table 1. White patients (79.56%) and colon cancers (69.56%) accounted for most of the cases in the present study. Patients with SRCC were slightly younger and more likely to be diagnosed with poorly differentiated or undifferentiated cancers, and a distant stage than the other subtypes. Most patients underwent surgical treatment for CRC: 95.51, 98.32, and 93.33% for patients with CA, MA, and SRCC, respectively. Patients with SRCC presented a higher ratio of receiving radiotherapy (16.41 vs. 14.87 vs. 21.39%, CA vs. MA vs. SRCC, respectively) and chemotherapy (44.74 vs. 51.07 vs. 67.81%, CA vs. MA vs. SRCC, respectively) than the other subtypes.

**Table 1:** Clinicopathological characteristics of patients with colorectal cancer stratified by histological subtypes.

**Variable**	**CA**	**MA**	**SRCC**	***p***
	***n* = 188,975 (%)**	***n* = 18,092 (%)**	***n* = 1,814 (%)**	
**Sex**				
Male	103,474 (54.76)	9,484 (52.42)	1,001 (55.18)	
Female	85,501 (45.24)	8,608 (47.58)	813(44.82)	<0.001
**Race**				
White	150,007 (79.38)	14,696 (81.23)	1,481 (81.64)	
Black	22,318 (11.81)	2,187 (12.09)	167 (9.21)	
Other	16,650 (8.81)	1,209 (6.68)	166 (9.15)	<0.001
**Age at diagnosis**				
20–49	25,950 (13.73)	2,812 (15.54)	475 (26.19)	
50–64	74,184 (39.26)	6,277 (34.69)	633 (34.9)	
65–79	88,841 (47.01)	9,003 (49.76)	706 (38.92)	<0.001
**Year of diagnosis**				
2000–2004	79,212 (41.92)	8,885 (49.11)	795 (43.83)	
2005–2009	79,155 (41.89)	6,907 (38.18)	743 (40.96)	
2010–2011	30,608 (16.2)	2,300 (12.71)	276 (15.21)	<0.001
**Grade**				
Well-differentiated; Grade I	18,992 (10.05)	2,044 (11.3)	17 (0.94)	
Moderately differentiated; Grade II	138,567 (73.33)	11,929 (65.94)	133 (7.33)	
Poorly differentiated; Grade III	29,446 (15.58)	3,781 (20.9)	1,515 (83.52)	
Undifferentiated; anaplastic; Grade IV	1,970 (1.04)	338 (1.87)	149 (8.21)	<0.001
**Site**				
Right colon	67,977 (35.97)	10,339 (57.15)	1,016 (56.01)	
Left colon	61,537 (32.56)	4,100 (22.66)	338 (18.63)	
Rectum	59,461 (31.47)	3,653 (20.19)	460 (25.36)	<0.001
**Stage**				
Distant	29,070 (15.38)	3,177 (17.56)	551 (30.37)	
Localized	79,540 (42.09)	5,179 (28.63)	207 (11.41)	
Regional	80,365 (42.53)	9,736 (53.81)	1,056 (58.21)	<0.001
**Surgery**				
No	8,485 (4.49)	304 (1.68)	121 (6.67)	
Yes	180,490 (95.51)	17,788 (98.32)	1,693 (93.33)	<0.001
**Radiotherapy**				
No/Unknown	157,957 (83.59)	15,402 (85.13)	1,426 (78.61)	
Yes	31,018 (16.41)	2,690 (14.87)	388 (21.39)	<0.001
**Chemotherapy**				
No/Unknown	104,420 (55.26)	8,852 (48.93)	584 (32.19)	
Yes	84,555 (44.74)	9,240 (51.07)	1,230 (67.81)	<0.001

*CA, classical adenocarcinoma; MA, mucinous adenocarcinoma; SRCC, signet-ring cell carcinoma*.

### Risks of Second Primary Malignancies

**Figure 1:**
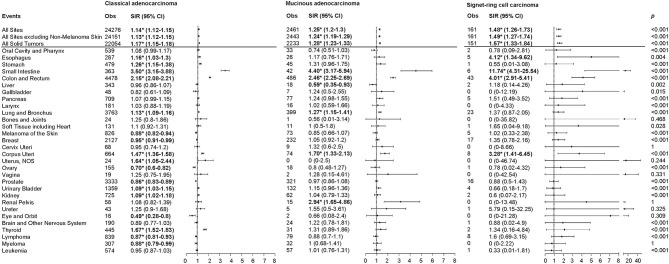
SIRs for SPMs at various anatomical sites based on the histological subtypes of the first primary CRC. Obs, observed events; SIR, standard incidence ratio; CI, confidence interval. *P < 0.05 (compared with general population). *P*-values comparing SIR differences among colorectal cancer subtypes were calculated using Poisson regression.

The overall risk of SPMs was higher among CRC patients than in the general population for the three CRC subtypes, with higher risks observed in MA and SRCC (CA: Obs = 24276, SIR = 1.14, 95% CI, 1.12-1.15; MA: Obs = 2461, SIR = 1.25, 95% CI, 1.2-1.3; SRCC: Obs = 161, SIR = 1.48, 95% CI, 1.26–1.73; *p* < 0.001; Figure 1). Notably, the risk patterns differed substantially among the three CRC subtypes. The risk of esophagus cancer was significantly increased in SRCC (SIR: CA = 1.16, MA = 1.17, SRCC = 4.12; *p* = 0.004), while the risk of lung and bronchus cancers was significantly increased in CA and MA, but not in SRCC. Risks of small intestine, colon and rectum, and corpus uteri cancers were increased in the three CRC subtypes, with the highest risk observed in SRCC, followed by MA (SIR for small intestine cancer: CA = 3.50, MA = 4.40, SRCC = 11.74; SIR for colon and rectum cancer: CA = 2.15, MA = 2.46, SRCC = 4.01; SIR for corpus uteri cancer: CA = 1.47, MA = 1.70, SRCC = 3.28; *p* < 0.001). Increased risk of renal pelvis cancer (SIR = 2.94) and reduced risk of liver cancer (SIR = 0.59) were limited to MA patients. In addition to the previously-mentioned cancers, the observed numbers of other second primary malignancies did not differ from expectation after MA and SRCC. However, CA patients were more likely to develop stomach (SIR = 1.26), uterus (SIR = 1.64), urinary bladder (SIR = 1.09), kidney (SIR = 1.09), and thyroid (SIR = 1.67) cancers, whereas less likely to develop melanoma of the skin (SIR = 0.88), breast (SIR = 0.95), ovary (SIR = 0.70), prostate (SIR = 0.86), eye and obit (SIR = 0.49), lymphoma (SIR = 0.87), and myeloma (SIR = 0.88) cancers.

**Figure 2:**
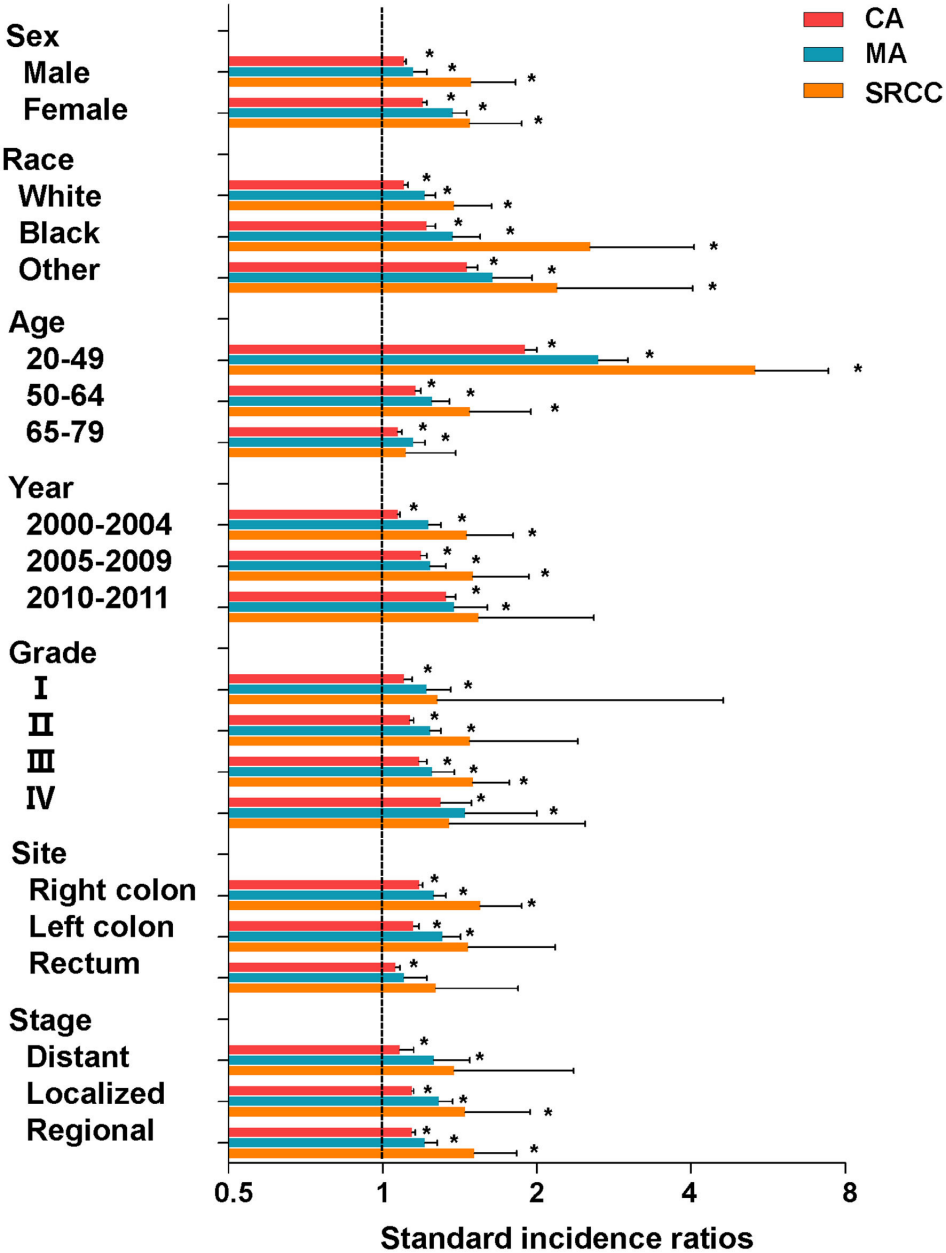
SIRs for SPMs at all anatomical sites combined among CRC survivors stratified by sex, race, age, year, grade, site, stage, and histological subtype. CA, classical adenocarcinoma; MA, mucinous adenocarcinoma; SRCC, signet-ring cell carcinoma. *P < 0.05 (compared with general population).

Furthermore, we conducted subgroup analyses of the overall risk of SPMs based on clinicopathological characteristics. The overall SIRs of CA patients remained high when stratified by different variables (Figure 2, Table 2). The overall SIRs of MA patients remained high in all subgroups other than that of rectum cancer (Table 2). Moreover, the overall SIRs of SRCC patients remained high when stratified by sex and race, but did not alter in several other subgroups (Table 2). Notably, among the three CRC subtypes, patients aged 20–49 years were more likely to develop SPMs than the older patients (Figure 2, Table 2).

**Table 2:** SIRs for SPMs at all anatomical sites combined among CRC survivors stratified by sex, race, age, year, grade, site, stage, and histological subtype.

	**CA**			**MA**			**SRCC**		
	**Obs**	**SIR**	**95% CI**	**Obs**	**SIR**	**95% CI**	**Obs**	**SIR**	**95% CI**
**Sex**									
Male	14,679	1.10^*^	1.08–1.11	1,342	1.15^*^	1.09–1.22	93	1.49^*^	1.2–1.82
Female	9,597	1.20^*^	1.18–1.22	1,119	1.37^*^	1.3–1.46	68	1.48^*^	1.15–1.87
**Race**									
White	19,895	1.10^*^	1.09–1.12	2,062	1.21^*^	1.16–1.27	134	1.38^*^	1.15–1.63
Black	2,704	1.22^*^	1.18–1.27	276	1.37^*^	1.22–1.55	17	2.54^*^	1.48–4.06
Other	1,677	1.46^*^	1.39–1.53	123	1.64^*^	1.36–1.96	10	2.19^*^	1.05–4.03
**Age at diagnosis**									
20–49	1,630	1.90^*^	1.81–2	220	2.64^*^	2.3–3.01	35	5.33^*^	3.71–7.41
50–64	8,501	1.16^*^	1.14–1.19	760	1.25^*^	1.17–1.35	50	1.48^*^	1.1–1.95
65–79	14,145	1.07^*^	1.05–1.09	1,481	1.15^*^	1.09–1.21	76	1.11	0.88–1.39
**Year of diagnosis**									
2000–2004	12,571	1.07^*^	1.05–1.08	1,477	1.23^*^	1.17–1.3	87	1.46^*^	1.17–1.8
2005–2009	9,273	1.19^*^	1.17–1.22	805	1.24^*^	1.16–1.33	60	1.50^*^	1.14–1.93
2010–2011	2,432	1.33^*^	1.28–1.39	179	1.38^*^	1.19–1.6	14	1.54	0.84–2.58
**Grade**									
I	2,821	1.10^*^	1.06–1.14	320	1.22^*^	1.09–1.36	2	1.28	0.15–4.62
II	17,926	1.13^*^	1.11–1.15	1,683	1.24^*^	1.19–1.3	16	1.48	0.84–2.4
III	3,322	1.18^*^	1.14–1.22	421	1.25^*^	1.14–1.38	133	1.50^*^	1.25–1.77
IV	207	1.30^*^	1.13–1.49	37	1.45^*^	1.02–2	10	1.35	0.65–2.48
**Site**									
Right colon	9,528	1.18^*^	1.16–1.2	1,514	1.26^*^	1.2–1.33	109	1.55^*^	1.27–1.87
Left colon	8,115	1.15^*^	1.13–1.18	568	1.31^*^	1.2–1.42	25	1.47	0.95–2.17
Rectum	6,633	1.06^*^	1.03–1.08	379	1.1	0.99–1.22	27	1.27	0.83–1.84
**Stage**									
Distant	1,145	1.08^*^	1.02–1.15	150	1.26^*^	1.06–1.48	13	1.38	0.73–2.36
Localized	13,212	1.14^*^	1.12–1.15	1,065	1.29^*^	1.21–1.37	44	1.45^*^	1.05–1.94
Regional	9,919	1.14^*^	1.12–1.16	1,246	1.21^*^	1.14–1.28	104	1.51^*^	1.24–1.83

CA, classical adenocarcinoma; MA, mucinous adenocarcinoma; SRCC, signet-ring cell carcinoma; Obs, observed events; SIR, standard incidence ratio; CI, confidence interval;

* *P < 0.05 (compared with general population)*.

### Risk of Second Primary Malignancies After Treatment

We further analyzed the risks of SPMs among the three histological subtypes of CRC after administration of different treatments. For CRC patients, we compared SIRs between surgery only and surgery combined with chemotherapy, since most patients received the two treatments. No statistically significant difference was observed in the overall SIRs of SPMs between the two treatment groups for the three CRC subtypes (CA, *p* = 0.445; MA, *p* = 0.421; SRCC, *p* = 0.209; Table 3, Supplementary Tables 2, 3). However, surgery combined with chemotherapy appeared to increase the risk of small intestine (surgery only group: SIR = 2.91; surgery combined with chemotherapy group: SIR = 5.62; *p* = 0.011), colon and rectum cancers (surgery only group: SIR = 2.07; surgery combined with chemotherapy group: SIR = 2.45; *p* = 0.009) in CA patients (Table 3).

**Table 3:** SPM risks at various anatomical sites based on previous surgery and chemotherapy treatments in colorectal CA patients.

**Events**	**Treatment for colorectal cancer**						***P***
	**Surgery only**			**Surgery combined with chemotherapy**			
	**Obs**	**SIR**	**95% CI**	**Obs**	**SIR**	**95% CI**	
All sites	15,449	1.15^*^	1.13–1.17	5,561	1.17^*^	1.14–1.2	0.445
All sites excluding non-melanoma skin	15,364	1.15^*^	1.13–1.17	5,530	1.17^*^	1.14–1.2	0.463
All solid tumors	14,000	1.18^*^	1.16–1.2	5,116	1.21^*^	1.18–1.24	0.286
Oral cavity and pharynx	357	1.17^*^	1.05–1.29	93	0.83	0.67–1.01	0.031^#^
Esophagus	196	1.26^*^	1.09–1.45	53	1	0.75–1.3	0.29
Stomach	330	1.36^*^	1.22–1.52	98	1.19	0.97–1.45	0.444
Small intestine	190	2.91^*^	2.51–3.36	131	5.62^*^	4.7–6.67	0.011^#^
Colon and rectum	2,760	2.07^*^	1.99–2.15	1116	2.45^*^	2.31–2.6	0.009^#^
Liver	229	1.05	0.92–1.2	68	0.84	0.65–1.07	0.237
Gallbladder	34	0.89	0.62–1.25	9	0.7	0.32–1.33	0.621
Pancreas	466	1.09	1–1.2	159	1.11	0.95–1.3	0.894
Larynx	110	1.01	0.83–1.22	44	1.13	0.82–1.51	0.68
Lung and bronchus	2,410	1.13^*^	1.08–1.17	770	1.06	0.99–1.14	0.319
Bones and joints	6	0.51	0.19–1.1	9	2.07	0.94–3.92	0.059
Soft tissue including heart	81	1.08	0.86–1.35	26	0.99	0.65–1.45	0.781
Melanoma of the skin	528	0.90^*^	0.82–0.98	178	0.87	0.75–1.01	0.809
Breast	1,364	0.99	0.93–1.04	500	0.90^*^	0.82–0.98	0.185
Cervix uteri	42	1	0.72–1.35	20	1.03	0.63–1.6	0.93
Corpus uteri	361	1.31^*^	1.18–1.45	183	1.58^*^	1.36–1.82	0.193
Uterus, NOS	9	0.96	0.44–1.82	6	1.71	0.63–3.72	0.474
Ovary	96	0.69^*^	0.56–0.84	48	0.89	0.66–1.18	0.28
Vagina	7	0.71	0.29–1.47	6	1.66	0.61–3.62	0.299
Prostate	2,301	0.97	0.93–1.01	808	0.96	0.9–1.03	0.849
Urinary bladder	861	1.06	0.99–1.13	278	1.08	0.96–1.22	0.83
Kidney	443	1.08	0.98–1.19	175	1.17^*^	1–1.36	0.553
Renal pelvis	35	0.99	0.69–1.38	13	1.16	0.62–1.99	0.735
Ureter	22	0.97	0.61–1.47	13	1.84	0.98–3.14	0.243
Eye and orbit	11	0.54^*^	0.27–0.97	3	0.42	0.09–1.22	0.733
Brain and other nervous system	119	0.9	0.75–1.08	41	0.86	0.61–1.16	0.836
Thyroid	235	1.52^*^	1.33–1.73	134	1.96^*^	1.64–2.32	0.158
Lymphoma	506	0.83^*^	0.76–0.9	187	0.89	0.77–1.03	0.544
Myeloma	222	1	0.87–1.14	47	0.62^*^	0.45–0.82	0.019^#^
Leukemia	376	0.97	0.87–1.07	110	0.85	0.7–1.03	0.392

CA, classical adenocarcinoma; Obs, observed events; SIR, standard incidence ratio; CI, confidence interval;

* *P < 0.05 (compared with general population)*.

# *P < 0.05. P-values comparing SIRs for colorectal CA survivors who received surgery only vs. surgery combined with chemotherapy were calculated using Poisson regression*.

In addition, we compared SIRs between surgery only and surgery combined with radiotherapy treatments in rectal cancer patients. Rectal SRCC patients were not included in this analysis due to the limited number of observed events of SPMs. No significant differences in the overall risk of SPMs in rectal CA and MA patients were observed between the two treatments (CA, *p* = 0.782; MA, *p* = 0.099; Table 4, Supplementary Table 4). However, a lower risk of second prostate cancer was observed in rectal CA patients of the surgery combined with radiotherapy group (surgery only group, SIR = 0.86, surgery combined with radiotherapy group, SIR = 0.26, *p* < 0.001; Table 4).

**Table 4:** SPM risks at various anatomical sites based on previous surgery and radiotherapy treatments in rectal CA patients.

**Events**	**Treatment for rectal cancer**						***p***
	**Surgery only**			**Surgery combined with radiotherapy**			
	**Obs**	**SIR**	**95% CI**	**Obs**	**SIR**	**95% CI**	
All sites	3,253	1.13^*^	1.1–1.17	213	1.1	0.96–1.26	0.782
All sites excluding non-melanoma skin	3,236	1.13^*^	1.09–1.17	213	1.11	0.96–1.27	0.821
All solid tumors	2,951	1.16^*^	1.12–1.21	192	1.12	0.97–1.29	0.742
Oral cavity and pharynx	74	1.08	0.85–1.36	4	0.86	0.23–2.21	0.745
Esophagus	47	1.38^*^	1.02–1.84	2	0.86	0.1–3.09	0.625
Stomach	57	1.12	0.85–1.45	2	0.58	0.07–2.09	0.459
Small Intestine	30	2.21^*^	1.49–3.16	4	4.40^*^	1.2–11.27	0.547
Colon and rectum	680	2.45^*^	2.27–2.64	33	1.74^*^	1.2–2.45	0.262
Liver	55	1.14	0.86–1.49	2	0.61	0.07–2.2	0.488
Gallbladder	7	0.93	0.37–1.91	0	0	0–7.49	0.428
Pancreas	81	0.92	0.73–1.15	4	0.69	0.19–1.76	0.657
Larynx	21	0.88	0.54–1.34	0	0	0–2.2	0.151
Lung and bronchus	504	1.13^*^	1.03–1.23	51	1.70^*^	1.26–2.23	0.085
Bones and joints	4	1.54	0.42–3.95	2	11.48^*^	1.39–41.48	0.343
Soft tissue including heart	19	1.18	0.71–1.85	0	0	0–3.43	0.201
Melanoma of the skin	113	0.86	0.71–1.03	14	1.61	0.88–2.7	0.156
Breast	239	0.86^*^	0.75–0.97	23	1.33	0.85–2	0.18
Cervix uteri	9	1.03	0.47–1.96	1	1.78	0.05–9.93	0.747
Corpus uteri	72	1.27	0.99–1.6	7	2.02	0.81–4.17	0.486
Uterus, NOS	0	0	0–2.12	0	0	0–33.98	0.999
Ovary	24	0.87	0.55–1.29	1	0.58	0.01–3.24	0.754
Vagina	2	1.07	0.13–3.88	1	8.65	0.22–48.21	0.445
Prostate	459	0.86^*^	0.78–0.94	10	0.26^*^	0.13–0.48	<0.001^#^
Urinary bladder	189	1.08	0.93–1.25	12	1.01	0.52–1.76	0.869
Kidney	89	1	0.8–1.23	3	0.5	0.1–1.46	0.321
Renal pelvis	8	1.08	0.47–2.13	2	4.06	0.49–14.65	0.385
Ureter	4	0.84	0.23–2.14	1	3.11	0.08–17.35	0.508
Eye and orbit	0	0.00^*^	0–0.82	0	0	0–12.41	0.999
Brain and other nervous system	25	0.86	0.56–1.27	1	0.51	0.01–2.87	0.678
Thyroid	47	1.34	0.98–1.78	2	0.89	0.11–3.2	0.677
Lymphoma	101	0.77^*^	0.63–0.94	9	1.04	0.47–1.97	0.554
Myeloma	37	0.82	0.58–1.13	3	0.98	0.2–2.87	0.83
Leukemia	85	1.03	0.83–1.28	4	0.73	0.2–1.87	0.602

CA, classical adenocarcinoma; Obs, observed events; SIR, standard incidence ratio; CI, confidence interval;

* *P < 0.05 (compared with general population)*.

# *P < 0.05. P-values comparing SIRs for rectal CA survivors who received surgery only vs. surgery combined with radiotherapy were calculated using Poisson regression*.

## Discussion

In the present large population-based study, we demonstrated for the first time that the risk of developing SPMs among CRC patients varied with the histological subtypes of CRC. Compared with the general population, the overall risk of SPMs was higher among CRC patients, which is consistent with previous study (3–7). However, when stratified by histological subtypes, we established that the overall risk of SPMs was slightly higher in MA and SRCC patients than in CA patients, while increased risk of SPMs in specific anatomical sites was observed more in CA patients. In addition, a significant increase in SIRs was only persistent in CA patients when stratified by different variables. The mechanism underlying the pattern is unclear, but it could be associated with the biological variations between the different subtypes of CRC. Indeed, CRC exhibits notable differences in incidence, location of tumor, pathogenesis, molecular pathways, and outcome based on the histological subtypes (17–19). Critical confounders such as lifestyle factors, genetic susceptibility, and detailed treatment data could also considerably influence the development of SPMs after CRC.

Patterns of the risk of SPMs after CRC indicate the existence of several overlapping mechanisms, including shared aetiological factors with the primary cancer, genetic predisposition and late side effects of cancer treatment (14, 15). Analysis of specific SPMs based on histological subtypes allows us to further explore the potential mechanisms facilitating the development of SPMs after CRC. For example, the present study revealed that the risk of second esophagus cancer was increased in CA and SRCC, and the risk of second lung and bronchus cancer was increased in CA and MA. This is likely associated with the well-established link between tobacco smoking and increased risk of CRC (20, 21). Similarly, increased risk of second kidney cancer in CA and second corpus uteri cancer in the three subtypes of CRC could partially be associated with obesity (22, 23).

Lynch syndrome is caused by germline mutations and is associated with an increased risk of colorectum, stomach, small intestine, and pancreatic cancers, as well as other genitourinary cancers (24–27). Similarly, hereditary non-polyposis CRC, which is attributed to mutations in the DNA mismatch repair genes, increases the risk of developing multiple primary CRCs and tumors at extracolonic sites, including the endometrium, ovary, small intestine, biliary tract, urinary tract, stomach, kidney, thyroid, and nervous system (16, 28, 29). In the present study, we established that specific risks of SPMs varied with the histological subtypes of CRC. For example, the risk of second stomach, uterus, urinary bladder, kidney, and thyroid cancers was only high in CA, while the risk of second renal pelvis cancer was specifically high in MA. However, the risk of second small intestine, colon and rectum, and corpus uteri cancers was high in the three subtypes of CRC. The increased risk of SPMs of specific anatomical sites in different histological subtypes of CRC necessitates the evaluation of family history and clinical screening for hereditary CRC.

Previous studies have revealed that young patients were more likely to develop SPMs (4, 30). Liang et al. demonstrated that young patients in Taiwan (aged <50 years) had a higher risk of developing SPMs than the general population (30). Furthermore, He et al. established that young CRC survivors exhibited a considerably high risk of developing SPMs in relation to the general population (4). In the present study, the significantly high risk of SPMs was observed in young patients aged 20–49 years in the three subtypes of CRC, which is consistent with the previous studies (4, 30). Although the mechanisms responsible for increased risk of SPMs among the younger population are poorly understood, a comprehensive understanding of the risk faced by young CRC patients and regular surveillance could help to determine appropriate prevention strategies.

Surgery is a standard therapy for curable CRC, and radiotherapy and chemotherapy are used as auxiliary therapies to a variable degree. MA patients appeared to be less responsive to chemotherapy, which could be partially explained by genetic differences such as high rates of microsatellite instability (31) and distinct patterns of tumor spread including peritoneal dissemination (32). Other studies have also revealed that MA patients exhibited a worse prognosis than non-MA patients despite of the different chemotherapy regimens being used (33–36). Conversely, Hugen et al. demonstrated that there was no significant interaction between SRCC and adjuvant chemotherapy when compared with CA, suggesting a comparable benefit from adjuvant chemotherapy in CA and SRCC (32). However, SRCC remained a poor prognostic factor when compared with CA, which could be due to its higher invasive potential (8, 10, 11, 32). Moreover, radiation and chemotherapy agents could contribute to the observed patterns of SPM risk, as a result of carcinogenic and immunosuppressive effects (14–16). Several studies have revealed an increased risk of CRC with chemotherapy, although the underlying mechanisms remain unknown (16, 37). In the present study, no significant difference in the overall risk of SPMs was observed between patients received surgery only and those received surgery combined with chemotherapy for the three subtypes of CRC. However, for CA patients, a higher risk of second small intestine, colon and rectum cancers was observed in the surgery combined with chemotherapy group than in the surgery only group. As mentioned above, a better overall survival in CA patients, which possessed them adequate time to develop SPMs, as well as the carcinogenic effects of chemotherapy, could play a role in the development of SPMs. Nevertheless, further investigation is required to understand the role of SPM risk caused by chemotherapy.

There is conflicting data on whether rectal cancer survivors are at high risk of developing SPMs due to radiotherapy. A few studies have reported an increased risk of SPMs after radiotherapy while others reported no increase or low risk (38–41). In the present study, no alteration of the overall risk of SPMs was observed after radiotherapy, but a reduced risk of second prostate cancer was observed in irradiated CA patients. The role of radiotherapy in CA patients in the present study is consistent with the findings of Martling et al., who reported that no increased risk of SPMs was observed in irradiated vs. non-irradiated patients; however, a reduced risk of prostate cancer was observed in irradiated patients (40). Moreover, a recent analysis of the Netherlands population-based cancer registry revealed that radiotherapy seemed to exhibit a protective effect against the development of other second pelvic tumors, predominantly prostate cancer, which is consistent with the results of the present study (41). By contrast, the Uppsala and Swedish Rectal Cancer Trials suggested an increased risk of SPMs in rectal cancer patients treated with radiotherapy in combination with surgery, which is contrary to the results of the present study (38). The contrasting observations could be explained by a few probable reasons. For example, most studies including the present study did not consider the irradiated volume and radiation dose received by patients, as well as the impact of preoperative or postoperative adjuvant radiotherapy. Furthermore, the studies did not investigate the impact of radiation based on the different histological subtypes and the sample sizes of most studies were small. In addition, the lower risk of prostate cancer after radiotherapy in CA patients observed in the present study could be partially attributed to “incidental” radiation of the prostate when treating the rectum. Hormonal changes caused by scattered radiation to the testicles during radiotherapy of the rectum could also impede the development of prostate cancer (42). Finally, the number of rectal cancer patients who developed SPMs after radiotherapy was small, which suggested that some observed associations could have occurred by chance. Therefore, we strongly recommend the use of a larger patient population with a considerable follow-up to draw firm conclusions with regard to the impact of radiation on rectal cancer patients based on the histological subtypes.

The key strength of the present study is an evaluation of SPM risks based on the histological subtypes of CRC. In addition, the use of large-scale population-based registry data enabled us to investigate the risk of developing SPMs among survivors of three specific histological subtypes of CRC. However, the present study had a few limitations. First, detailed information on lifestyle characteristics, family history, genetic factors, as well as specific treatment regiments could not be obtained from the database. Second, a diagnostic bias in CRC survivors could have existed, because the patients likely underwent more examinations and surveillance than the general population. Finally, despite of the large number of CRC survivors in the SEER database, cases used for SIR calculations were limited to less common SPM sites, particularly when stratified by histological subtypes of CRC.

## Conclusion

The present study revealed substantial differences in the risk of developing SPMs among specific CRC subtypes. Further studies with detailed patient medical history, treatment regiments, and laboratory data should be conducted to validate the results of the present study. Overall, the findings suggest that strategies for cancer surveillance after previous CRC could be personalized based on the histological subtype of previous CRC.

## Data Availability Statement

Publicly available datasets were analyzed in this study. This data can be found here: Surveillance, Epidemiology, and End Results (SEER) database (https://seer.cancer.gov/).

## Author Contributions

XC, ZL, and GF: study concept and design. MW: acquisition of data. MH and CH: analysis and interpretation of data. CC, HL, JW, and ML: critical revision of manuscript. All the authors were involved in approval of the final version.

## Conflict of Interest

The authors declare that the research was conducted in the absence of any commercial or financial relationships that could be construed as a potential conflict of interest.

## Supplementary Material

Supplementary Figure 1 A flow chart of the selection criteria of patients enrolled in the Surveillance, Epidemiology, and End Results (SEER) research database.
