# Supplementary material for: Risk of Second Primary Malignancies Based on the Histological Subtypes of Colorectal Cancer
Source: Front Oncol. 2021 Mar 10;11:650937. doi: 10.3389/fonc.2021.650937 (PMC7988191; doi:10.3389/fonc.2021.650937)
Supplement: Supplementary file 1 [file Data_Sheet_1.docx]

**Supplementary materials**

**Table S1. The number of enrolled patients with different histological subtypes of colorectal cancer.**

| **Histological subtype** | **ICD-O-3 code** | ***n*** |
| --- | --- | --- |
| **Classical adenocarcinoma** | 8140/3: Adenocarcinoma, NOS | 149416 |
|  | 8141/3: Scirrhous adenocarcinoma | 12 |
|  | 8143/3: Superficial spreading adenocarcinoma | 3 |
|  | 8144/3: Adenocarcinoma, intestinal type | 40 |
|  | 8147/3: Basal cell adenocarcinoma | 1 |
|  | 8210/3: Adenocarcinoma in adenomatous polyp | 14879 |
|  | 8211/3: Tubular adenocarcinoma | 137 |
|  | 8213/3: Serrated adenocarcinoma | 10 |
|  | 8220/3: Adenocarcinoma in adenomatous polyposis coli | 80 |
|  | 8221/3: Adenocarcinoma in multiple adenomatous polyps | 48 |
|  | 8255/3: Adenocarcinoma with mixed subtypes | 448 |
|  | 8260/3: Papillary adenocarcinoma, NOS | 71 |
|  | 8261/3: Adenocarcinoma in villous adenoma | 6360 |
|  | 8262/3: Villous adenocarcinoma | 189 |
|  | 8263/3: Adenocarcinoma in tubulovillous adenoma | 17254 |
|  | 8310/3: Clear cell adenocarcinoma, NOS | 6 |
|  | 8323/3: Mixed cell adenocarcinoma | 21 |
| **Mucinous adenocarcinoma** | 8480/3: Mucinous adenocarcinoma | 13902 |
|  | 8481/3: Mucin-producing adenocarcinoma | 4190 |
| **Signet-ring cell carcinoma** | 8490/3: Signet-ring cell carcinoma | 1814 |
| **Overall** |  | 208881 |

**Table S2. SPM risks at various anatomical sites based on previous surgery and chemotherapy treatments in colorectal MA patients.**

|  | **Treatment for colorectal cancer** | | | | | | |  |
| --- | --- | --- | --- | --- | --- | --- | --- | --- |
|  | **Surgery only** | | |  | **Surgery combined with chemotherapy** | | |  |
| **Events** | **Obs** | **SIR** | **95% CI** |  | **Obs** | **SIR** | **95% CI** | ***p*** |
| All Sites | 1433 | 1.25^*^ | 1.19-1.32 |  | 783 | 1.32^*^ | 1.23-1.42 | 0.421 |
| All Sites excluding Non-Melanoma Skin | 1421 | 1.25^*^ | 1.18-1.31 |  | 778 | 1.32^*^ | 1.23-1.41 | 0.41 |
| All Solid Tumors | 1296 | 1.29^*^ | 1.22-1.36 |  | 719 | 1.37^*^ | 1.27-1.47 | 0.386 |
| Oral Cavity and Pharynx | 16 | 0.64 | 0.37-1.04 |  | 16 | 1.15 | 0.66-1.87 | 0.229 |
| Esophagus | 14 | 1.09 | 0.6-1.83 |  | 11 | 1.67 | 0.83-2.99 | 0.494 |
| Stomach | 28 | 1.38 | 0.91-1.99 |  | 11 | 1.11 | 0.55-1.98 | 0.678 |
| Small Intestine | 26 | 4.68^*^ | 3.06-6.86 |  | 12 | 4.17^*^ | 2.15-7.28 | 0.885 |
| Colon and Rectum | 278 | 2.37^*^ | 2.1-2.66 |  | 163 | 2.83^*^ | 2.41-3.3 | 0.34 |
| Liver | 7 | 0.41^*^ | 0.17-0.85 |  | 8 | 0.84 | 0.36-1.66 | 0.279 |
| Gallbladder | 4 | 1.15 | 0.31-2.96 |  | 1 | 0.61 | 0.02-3.4 | 0.659 |
| Pancreas | 41 | 1.1 | 0.79-1.49 |  | 29 | 1.61^*^ | 1.08-2.31 | 0.305 |
| Larynx | 13 | 1.49 | 0.79-2.55 |  | 2 | 0.42 | 0.05-1.52 | 0.165 |
| Lung and Bronchus | 250 | 1.34^*^ | 1.18-1.52 |  | 105 | 1.15 | 0.94-1.39 | 0.357 |
| Bones and Joints | 0 | 0 | 0-3.67 |  | 1 | 1.82 | 0.05-10.17 | 0.235 |
| Soft Tissue including Heart | 3 | 0.47 | 0.1-1.38 |  | 5 | 1.53 | 0.5-3.57 | 0.229 |
| Melanoma of the Skin | 44 | 0.9 | 0.65-1.2 |  | 23 | 0.89 | 0.56-1.33 | 0.984 |
| Breast | 143 | 1.11 | 0.94-1.31 |  | 69 | 0.97 | 0.75-1.22 | 0.509 |
| Cervix Uteri | 6 | 1.6 | 0.59-3.48 |  | 2 | 0.85 | 0.1-3.06 | 0.584 |
| Corpus Uteri | 30 | 1.2 | 0.81-1.71 |  | 32 | 2.21^*^ | 1.51-3.13 | 0.139 |
| Uterus, NOS | 0 | 0 | 0-4.04 |  | 0 | 0 | 0-8.26 | 0.999 |
| Ovary | 11 | 0.82 | 0.41-1.47 |  | 6 | 0.85 | 0.31-1.85 | 0.962 |
| Vagina | 1 | 1.04 | 0.03-5.77 |  | 1 | 2.1 | 0.05-11.7 | 0.753 |
| Prostate | 177 | 0.97 | 0.83-1.13 |  | 120 | 1.2 | 0.99-1.43 | 0.225 |
| Urinary Bladder | 81 | 1.18 | 0.94-1.47 |  | 34 | 1.06 | 0.73-1.48 | 0.716 |
| Kidney | 36 | 1.07 | 0.75-1.48 |  | 19 | 1.03 | 0.62-1.61 | 0.935 |
| Renal Pelvis | 8 | 2.58^*^ | 1.11-5.08 |  | 6 | 4.19^*^ | 1.54-9.13 | 0.667 |
| Ureter | 3 | 1.52 | 0.31-4.44 |  | 1 | 1.11 | 0.03-6.2 | 0.855 |
| Eye and Orbit | 2 | 1.16 | 0.14-4.2 |  | 0 | 0 | 0-4.02 | 0.272 |
| Brain and Other Nervous System | 16 | 1.43 | 0.81-2.31 |  | 6 | 0.99 | 0.36-2.16 | 0.601 |
| Thyroid | 15 | 1.21 | 0.67-1.99 |  | 10 | 1.22 | 0.59-2.24 | 0.985 |
| Lymphoma | 47 | 0.89 | 0.65-1.19 |  | 17 | 0.64 | 0.37-1.02 | 0.365 |
| Myeloma | 23 | 1.2 | 0.76-1.81 |  | 7 | 0.74 | 0.3-1.53 | 0.408 |
| Leukemia | 31 | 0.92 | 0.63-1.31 |  | 21 | 1.28 | 0.79-1.96 | 0.423 |

Abbreviations: MA, mucinous adenocarcinoma; Obs, observed events; SIR, standard incidence ratio; CI, confidence interval; ^*^*P* < 0.05 (compared with general population). *P* values comparing SIRs for colorectal MA survivors who received surgery only versus surgery combined with chemotherapy were calculated using Poisson regression.

**Table S3. SPM risks at various anatomical sites based on previous surgery and chemotherapy treatments in colorectal SRCC patients.**

|  | **Treatment for colorectal cancer** | | | | | | |  |
| --- | --- | --- | --- | --- | --- | --- | --- | --- |
|  | **Surgery only** | | |  | **Surgery combined with chemotherapy** | | |  |
| **Events** | **Obs** | **SIR** | **95% CI** |  | **Obs** | **SIR** | **95% CI** | ***p*** |
| All Sites | 64 | 1.29 | 0.99-1.64 |  | 75 | 1.80^*^ | 1.42-2.26 | 0.209 |
| All Sites excluding Non-Melanoma Skin | 64 | 1.29 | 0.99-1.65 |  | 75 | 1.81^*^ | 1.42-2.27 | 0.211 |
| All Solid Tumors | 61 | 1.39^*^ | 1.07-1.79 |  | 68 | 1.84^*^ | 1.43-2.34 | 0.323 |
| Oral Cavity and Pharynx | 0 | 0 | 0-3.39 |  | 2 | 2.01 | 0.24-7.25 | 0.174 |
| Esophagus | 2 | 3.73 | 0.45-13.46 |  | 1 | 2.17 | 0.05-12.06 | 0.821 |
| Stomach | 0 | 0 | 0-4.39 |  | 1 | 1.5 | 0.04-8.36 | 0.289 |
| Small Intestine | 1 | 4.25 | 0.11-23.69 |  | 3 | 15.34^*^ | 3.16-44.82 | 0.694 |
| Colon and Rectum | 14 | 2.75^*^ | 1.5-4.61 |  | 24 | 6.03^*^ | 3.87-8.98 | 0.291 |
| Liver | 2 | 2.79 | 0.34-10.07 |  | 0 | 0 | 0-5.88 | 0.241 |
| Gallbladder | 0 | 0 | 0-23.82 |  | 0 | 0 | 0-33.48 | 0.999 |
| Pancreas | 5 | 3.09^*^ | 1-7.21 |  | 0 | 0 | 0-3 | 0.088 |
| Larynx | 0 | 0 | 0-10.34 |  | 0 | 0 | 0-11.18 | 0.999 |
| Lung and Bronchus | 11 | 1.36 | 0.68-2.44 |  | 8 | 1.27 | 0.55-2.5 | 0.92 |
| Bones and Joints | 0 | 0 | 0-82.39 |  | 0 | 0 | 0-91.73 | 1 |
| Soft Tissue including Heart | 0 | 0 | 0-13.43 |  | 0 | 0 | 0-15.82 | 0.999 |
| Melanoma of the Skin | 2 | 0.92 | 0.11-3.32 |  | 3 | 1.54 | 0.32-4.5 | 0.702 |
| Breast | 9 | 1.45 | 0.66-2.76 |  | 6 | 1.21 | 0.44-2.63 | 0.816 |
| Cervix Uteri | 0 | 0 | 0-20.46 |  | 0 | 0 | 0-20.16 | 1 |
| Corpus Uteri | 4 | 3.43 | 0.94-8.79 |  | 3 | 3.04 | 0.63-8.89 | 0.934 |
| Uterus, NOS | 0 | 0 | 0-88.91 |  | 0 | 0 | 0-123.68 | 0.999 |
| Ovary | 0 | 0 | 0-5.62 |  | 1 | 2.03 | 0.05-11.31 | 0.298 |
| Vagina | 0 | 0 | 0-79.39 |  | 0 | 0 | 0-115.29 | 0.999 |
| Prostate | 6 | 0.82 | 0.3-1.79 |  | 8 | 1.13 | 0.49-2.22 | 0.673 |
| Urinary Bladder | 2 | 0.71 | 0.09-2.56 |  | 2 | 0.88 | 0.11-3.17 | 0.874 |
| Kidney | 0 | 0 | 0-2.55 |  | 2 | 1.55 | 0.19-5.58 | 0.152 |
| Renal Pelvis | 0 | 0 | 0-27.15 |  | 0 | 0 | 0-36.71 | 0.999 |
| Ureter | 1 | 11.66 | 0.3-64.95 |  | 0 | 0 | 0-58.45 | 0.604 |
| Eye and Orbit | 0 | 0 | 0-47.46 |  | 0 | 0 | 0-54.11 | 0.999 |
| Brain and Other Nervous System | 1 | 1.98 | 0.05-11.05 |  | 0 | 0 | 0-8.33 | 0.378 |
| Thyroid | 0 | 0 | 0-6.37 |  | 1 | 1.56 | 0.04-8.69 | 0.353 |
| Lymphoma | 3 | 1.28 | 0.26-3.75 |  | 5 | 2.63 | 0.86-6.15 | 0.553 |
| Myeloma | 0 | 0 | 0-4.69 |  | 0 | 0 | 0-5.91 | 0.999 |
| Leukemia | 0 | 0 | 0-2.53 |  | 1 | 0.86 | 0.02-4.8 | 0.257 |

Abbreviations: SRCC, signet-ring cell carcinoma; Obs, observed events; SIR, standard incidence ratio; CI, confidence interval; ^*^*P* < 0.05 (compared with general population). *P* values comparing SIRs for colorectal SRCC survivors who received surgery only versus surgery combined with chemotherapy were calculated using Poisson regression.

**Table S4. SPM risks at various anatomical sites based on previous surgery and radiotherapy treatments in rectal MA patients.**

|  | **Treatment for rectal cancer** | | | | | | |  |
| --- | --- | --- | --- | --- | --- | --- | --- | --- |
|  | **Surgery only** | | |  | **Surgery combined with radiotherapy** | | |  |
| **Events** | **Obs** | **SIR** | **95% CI** |  | **Obs** | **SIR** | **95% CI** | ***p*** |
| All Sites | 128 | 1.16 | 0.97-1.38 |  | 5 | 0.48 | 0.15-1.11 | 0.099 |
| All Sites excluding Non-Melanoma Skin | 127 | 1.16 | 0.96-1.37 |  | 5 | 0.48 | 0.16-1.12 | 0.102 |
| All Solid Tumors | 119 | 1.22^*^ | 1.01-1.46 |  | 5 | 0.54 | 0.17-1.25 | 0.14 |
| Oral Cavity and Pharynx | 3 | 1.15 | 0.24-3.35 |  | 0 | 0 | 0-14.73 | 0.543 |
| Esophagus | 1 | 0.74 | 0.02-4.14 |  | 0 | 0 | 0-28.69 | 0.709 |
| Stomach | 3 | 1.53 | 0.32-4.48 |  | 0 | 0 | 0-20.03 | 0.569 |
| Small Intestine | 1 | 1.93 | 0.05-10.74 |  | 0 | 0 | 0-74.83 | 0.747 |
| Colon and Rectum | 29 | 2.62^*^ | 1.76-3.77 |  | 1 | 0.95 | 0.02-5.27 | 0.483 |
| Liver | 1 | 0.57 | 0.01-3.19 |  | 1 | 5.87 | 0.15-32.71 | 0.354 |
| Gallbladder | 1 | 3.45 | 0.09-19.24 |  | 1 | 37.84 | 0.96-210.84 | 0.643 |
| Pancreas | 2 | 0.59 | 0.07-2.12 |  | 0 | 0 | 0-11.71 | 0.594 |
| Larynx | 1 | 1.06 | 0.03-5.91 |  | 0 | 0 | 0-40.16 | 0.722 |
| Lung and Bronchus | 15 | 0.86 | 0.48-1.41 |  | 1 | 0.61 | 0.02-3.39 | 0.79 |
| Bones and Joints | 0 | 0 | 0-37.13 |  | 0 | 0 | 0-380.14 | 0.999 |
| Soft Tissue including Heart | 0 | 0 | 0-5.98 |  | 1 | 17.19 | 0.44-95.79 | 0.179 |
| Melanoma of the Skin | 6 | 1.17 | 0.43-2.55 |  | 0 | 0 | 0-8 | 0.405 |
| Breast | 14 | 1.48 | 0.81-2.49 |  | 0 | 0 | 0-4.06 | 0.205 |
| Cervix Uteri | 1 | 3.67 | 0.09-20.44 |  | 0 | 0 | 0-115.05 | 0.763 |
| Corpus Uteri | 0 | 0 | 0-2 |  | 0 | 0 | 0-20.81 | 0.999 |
| Uterus, NOS | 0 | 0 | 0-59.76 |  | 0 | 0 | 0-581.97 | 0.999 |
| Ovary | 0 | 0 | 0-3.76 |  | 0 | 0 | 0-39.97 | 0.999 |
| Vagina | 0 | 0 | 0-54.34 |  | 0 | 0 | 0-565.64 | 0.999 |
| Prostate | 22 | 1.03 | 0.65-1.56 |  | 0 | 0 | 0-1.75 | 0.088 |
| Urinary Bladder | 9 | 1.27 | 0.58-2.41 |  | 0 | 0 | 0-5.74 | 0.313 |
| Kidney | 5 | 1.47 | 0.48-3.44 |  | 0 | 0 | 0-11.27 | 0.446 |
| Renal Pelvis | 0 | 0 | 0-12.51 |  | 0 | 0 | 0-137.9 | 0.999 |
| Ureter | 0 | 0 | 0-19.28 |  | 0 | 0 | 0-210.92 | 0.999 |
| Eye and Orbit | 1 | 5.76 | 0.15-32.08 |  | 0 | 0 | 0-230.43 | 0.784 |
| Brain and Other Nervous System | 0 | 0 | 0-3.31 |  | 0 | 0 | 0-34.57 | 0.999 |
| Thyroid | 1 | 0.87 | 0.02-4.86 |  | 0 | 0 | 0-31.38 | 0.704 |
| Lymphoma | 3 | 0.59 | 0.12-1.73 |  | 0 | 0 | 0-7.82 | 0.516 |
| Myeloma | 0 | 0 | 0-2.1 |  | 0 | 0 | 0-22.08 | 0.999 |
| Leukemia | 2 | 0.62 | 0.07-2.22 |  | 0 | 0 | 0-12.42 | 0.598 |

Abbreviations: MA, mucinous adenocarcinoma; Obs, observed events; SIR, standard incidence ratio; CI, confidence interval; ^*^*P* < 0.05 (compared with general population). *P* values comparing SIRs for rectal MA survivors who received surgery only versus surgery combined with radiotherapy were calculated using Poisson regression.
