# Supplementary figures and images for: Risk of Second Primary Malignancies Based on the Histological Subtypes of Colorectal Cancer
Source: Front Oncol. 2021 Mar 10;11:650937. doi: 10.3389/fonc.2021.650937 (PMC7988191; doi:10.3389/fonc.2021.650937)

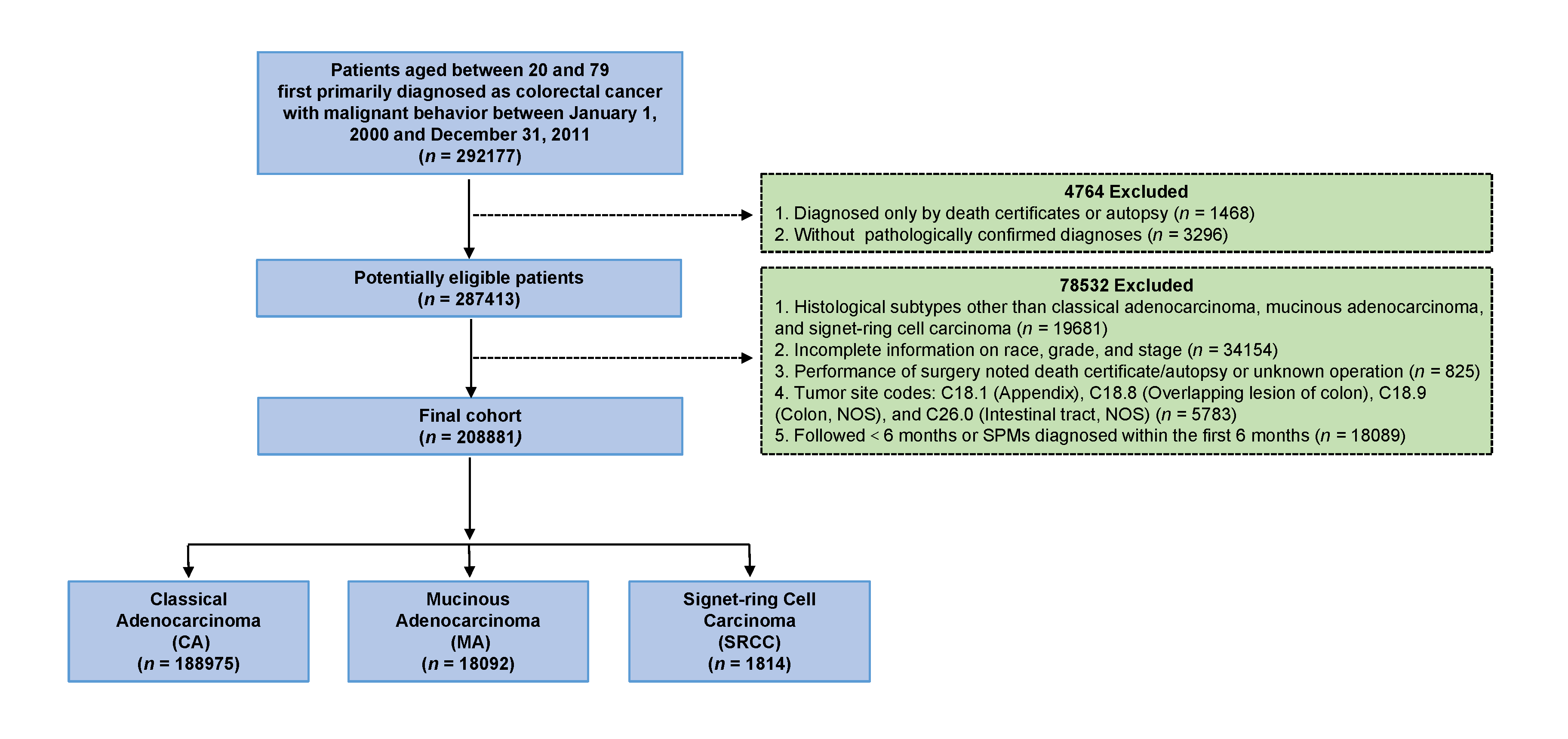

Supplement: Supplementary Figure 1 — A flow chart of the selection criteria of patients enrolled in the Surveillance, Epidemiology, and End Results (SEER) research database. [file Image_1.TIFF]
